# Quantum chemical calculations of lithium-ion battery electrolyte and interphase species

**DOI:** 10.1038/s41597-021-00986-9

**Published:** 2021-08-05

**Authors:** Evan Walter Clark Spotte-Smith, Samuel M. Blau, Xiaowei Xie, Hetal D. Patel, Mingjian Wen, Brandon Wood, Shyam Dwaraknath, Kristin Aslaug Persson

**Affiliations:** 1 University of California Berkeley, Department of Materials Science and Engineering, Berkeley, CA 94720 USA; 2 Lawrence Berkeley National Laboratory, Materials Science Division, Berkeley, CA 94720 USA; 3 Lawrence Berkeley National Laboratory, Energy Storage and Distributed Resources, Berkeley, CA 94720 USA; 4 University of California Berkeley, Department of Chemistry, Berkeley, CA 94720 USA; 5 Lawrence Berkeley National Laboratory, National Energy Research Supercomputing Center, Berkeley, CA 94720 USA; 6 Lawrence Berkeley National Laboratory, Molecular Foundry, Berkeley, CA 94720 USA

**Keywords:** Density functional theory, Energy, Batteries

## Abstract

Lithium-ion batteries (LIBs) represent the state of the art in high-density energy storage. To further advance LIB technology, a fundamental understanding of the underlying chemical processes is required. In particular, the decomposition of electrolyte species and associated formation of the solid electrolyte interphase (SEI) is critical for LIB performance. However, SEI formation is poorly understood, in part due to insufficient exploration of the vast reactive space. The Lithium-Ion Battery Electrolyte (LIBE) dataset reported here aims to provide accurate first-principles data to improve the understanding of SEI species and associated reactions. The dataset was generated by fragmenting a set of principal molecules, including solvents, salts, and SEI products, and then selectively recombining a subset of the fragments. All candidate molecules were analyzed at the *ω*B97X-V/def2-TZVPPD/SMD level of theory at various charges and spin multiplicities. In total, LIBE contains structural, thermodynamic, and vibrational information on over 17,000 unique species. In addition to studies of reactivity in LIBs, this dataset may prove useful for machine learning of molecular and reaction properties.

## Abstract (summary)

**Table:** 

Measurement(s)	molecule • solid electrolyte interphase
Technology Type(s)	density functional theory • computational modeling technique
Factor Type(s)	bond type • charge • spin multiplicity

Machine-accessible metadata file describing the reported data: 10.6084/m9.figshare.14915256

## Background & Summary

The solid electrolyte interphase (SEI), a nanoscale film that forms from electrolyte decomposition at the anodes of lithium-ion batteries (LIBs) during initial charging, is a critical component of modern rechargeable LIB electrolytes^1^. The SEI is responsible for the initial irreversible capacity loss during the battery’s first several charge-discharge cycles^2^. At the same time, an appropriate self-limiting SEI, once formed, can protect against continuous electrolyte degradation while allowing Li-ion conduction^3^. In spite of the SEI’s central importance to battery performance and lifespan, much remains unknown regarding the formation mechanisms of the SEI in LIBs. The SEI is formed as a result of numerous competitive reactive processes occurring simultaneously over time scales ranging from picoseconds^4^ to days^5^. As a result of this extreme complexity, there are many open questions regarding the reaction pathways involved and even the products that form along those pathways^6,7^.

It should be expected that many reactive intermediates that might arise during SEI formation will be so short-lived that they will be difficult or impossible to identify via experimental interrogations. However, even very reactive molecules can often be studied using first-principles quantum chemical methods such as density functional theory (DFT). Such computational simulations can therefore fill a gap and potentially provide a more fundamental understanding of the reactive chemistry of the SEI - for instance through the calculation of reaction free energies and energy barriers^8–12^. DFT can additionally be used to generate reference spectra that can be compared to experiment^13^.

Here, we describe **LIBE**, the **L**i-**I**on **B**attery **E**lectrolyte dataset. LIBE includes non-polymeric and non-oligomeric molecules relevant to SEI formation in LIBs, with molecular properties such as optimized geometries, molecular thermochemistry, and vibrational spectra calculated using DFT. These molecules, which include both species previously reported in the literature as well as many novel species, could form at the SEI as a result of electrolyte decomposition or the recombination of electrolyte fragments. The main purpose of LIBE is for studies of SEI formation and reactivity. Already, our group has used a subset of this data to generate a massive computational reaction network, identifying novel and chemically reasonable reactive pathways to a key SEI product, lithium ethylene dicarbonate (LEDC)^14^. It would be possible to take a similar approach to automatically identify pathways to other SEI products of interest, or perhaps to search for novel products not previously identified in experiments.

Far from being a single-use dataset of relevance only to SEI researchers, LIBE has the possibility of being used for broader studies of chemical reactions. For instance, the diverse molecules included in LIBE, including highly reactive and unstable species, provide an excellent dataset for machine learning models. We have recently used a subset of LIBE, which we called the “Bond Dissociation of Neutral and Charged Molecules” (BDNCM) dataset, to train a graph neural network called BonDNet^15^. BonDNet was able to predict heterolytic and homolytic bond dissociation energies with mean absolute error (MAE) far below chemical accuracy (0.022 eV vs. chemical accuracy of 0.043 eV).

**Fig. 1:**
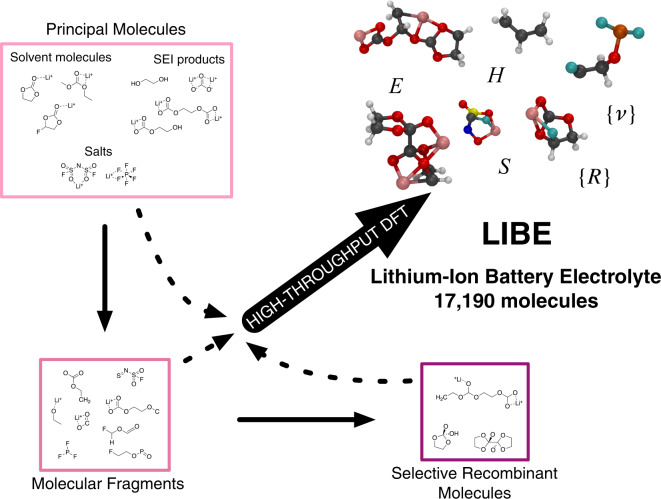
Overview of the process underlying the generation of the Lithium-Ion Battery Electrolyte (LIBE) dataset. A set of principal molecules relevant to LIB SEI formation, including solvent molecules, electrolytes, and SEI products, were first selected. These molecules were then broken up into fragments, and these fragments were allowed to selectively recombine to form new, larger molecules. All principal molecules, fragments, and recombinant molecules were analyzed using high-throughput DFT, which provides an understanding of their structure and atomic coordinates {R}, thermodynamics - including energy E, enthalpy H, and entropy S - and vibrational frequencies {ν}.

The remainder of this Data Descriptor is organized as follows: first, we describe the computational methods used to both generate a set of candidate molecules and calculate their properties using DFT (Fig. 1). We then explain the choices made in designing the dataset, including the computational level of theory and the filters applied to ensure the quality of the data. After this explanation, we briefly characterize the LIBE dataset, examining the types of molecular species that it contains in terms of elements, bond types, charge, and spin multiplicity, among other factors. Finally, we describe the codes used to generate and analyze LIBE, all of which are freely available in open source repositories.

## Methods

### Overview

Reactive organometallic molecules present significant challenges for computational analysis. Conventional methods to define molecular graph representations - necessary to define bonding and study molecular reactivity - are insufficient to capture coordinate bonds between Li^+^ and heavy atoms like O, F, and N. In addition, DFT calculations involving highly reactive charged, radical, and metal-coordinated molecules frequently encounter errors or fail to converge to stable potential energy surface minima. Methods to address both of these challenges are here described.

Data set construction is initialized with a small set of molecules known or previously proposed to participate in LIB SEI formation. From these “principal molecules”, a set of molecular fragments were generated by recursively breaking bonds in the molecular graph representations. To explore molecular formation beyond what is currently known, a subset of these molecular fragments was then recombined, adding bonds between fragments to create new molecules. Through the application of fragmentation and recombination methods, a collection of molecules were created that could connect initial electrolyte components to final SEI products, allowing for the exploration of the reactive chemistry of LIB electrolytes. All molecules were analyzed using DFT to produce optimized geometries, molecular thermodynamics (including energy, enthalpy, entropy, and free energy), and vibrational data (including calculated infrared spectra).

### Determination of bonding and molecular graph representations

**Fig. 2:**
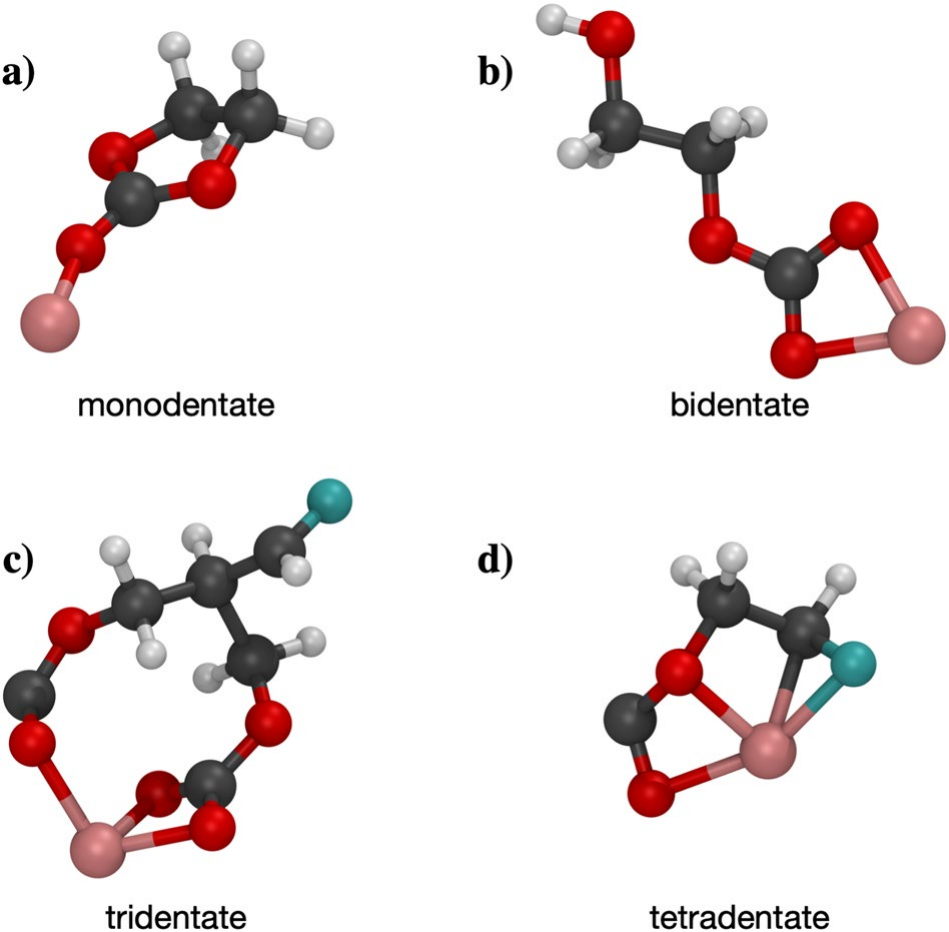
Examples of molecules with various Li^+^ coordination environments: monodentate (**a**), bidentate (**b**), tridentate (**c**), and tetradentate (**d**). White atoms are hydrogen, gray atoms are carbon, red atoms are oxygen, blue are fluorine, and pink are lithium.

Initially, bonding for all molecules was determined from 3D atomic coordinates using the bond detection algorithm defined in OpenBabel^16,17^. While this algorithm is well suited to the detection of covalent bonds, it is not designed to capture ionic bonds or coordinate bonds between metal ions and molecules^18^. Specifically, it is assumed in OpenBabel that Li^+^ will only form one bond. This is a critical issue for LIBE due to the crucially important and diverse coordination behavior of Li^+^. Li^+^ generally seeks to form between 4 and 6 coordinate bonds when in an electrolyte solution^13,19,20^. While often, Li^+^ forms only one coordinate bond with each coordinated molecule (Fig. 2a), cases where two (Fig. 2b), three (Fig. 2c), and even four (Fig. 2d) coordinate bonds form can occur. Because the thermodynamics of monodentate, bidentate, tridentate, and tetradentate configurations can vary significantly, it is essential to be able to distinguish between these bonding motifs. A modified bond detection algorithm was therefore required.

A heuristic method was used to add neglected coordinate bonds between Li and electronegative coordinating atoms, namely N, O, F, and S. If an N, O, F, or S atom is less than 2.5 away from a Li atom, then those two atoms were considered to be bonded. If, after this procedure, there were Li atoms in the molecule with no bonds, then the cutoff was increased from 2.5 to 3.5, and the procedure was repeated. Prior to performing DFT calculations, molecular connectivity was defined through first defining the bonding using OpenBabel and then applying this heuristic method.

In the final LIBE dataset, a quantum chemical method was also used to identify bonds. The Critic2 program^21,22^ was employed to identify bonding interactions in the electron densities of the optimized molecular geometry. Critic2 identifies critical points in the electron density, which correspond to interatomic interactions. If the calculated field at a critical point between two atoms is greater than 0.02 (in atomic units) and if the distance between atoms is less than 2.5, then the two atoms are considered to be bonded. An exception is made for bonds between Li and C, for which a smaller field (greater than 0.012) is allowed. The final bonding for a molecule was defined by the union of the sets of bonds identified using OpenBabel, the heuristic coordinate bond detection method, and Critic2.

### High-throughput computational methods

In order to be able to compute the properties of arbitrary molecules, including highly reactive fragments, radicals, and charged species, an automated framework was developed for high-throughput molecular DFT. This framework, which incorporates methods to correct common errors and ensure convergence to potential energy surface (PES) minima during molecular DFT calculations on the fly, was used to compute all properties of all molecules described in LIBE. Here, the computational methods used for high-throughput DFT calculations are described; an overview of how these methods were implemented in open source code bases is provided in Code Availability.

#### Calculation parameters

All calculations discussed in this Data Descriptor were performed using version 5.2.2 of the Q-Chem electronic structure code^23^. A large quadrature grid (SG-3) was used for all calculations^24^, and the cutoff for the neglect of two-electron integrals is set to the tightest possible value (10^−14^). Molecular symmetry was not used to improve calculation efficiency. Unless otherwise noted, with this exception, all Q-Chem default values (as of the 5.2.2 version) were used for initial calculations, though during error-correction these default values might be changed.

This dataset employs a level of theory based on the *ω*B97X-V density functional^25^, which leverages the VV10 nonlocal van der Waals density functional^26^ to accurately model noncovalent interactions. The def2-TZVPPD basis set^27,28^ is employed, and solvation effects were included implicitly by means of the SMD method^29^, which adds short-range energy contributions to the polarizable continuum model (PCM)^30,31^. The dielectric constant used (*ε* = 18.5) is that of a 3:7 ethylene carbonate (EC):ethyl methyl carbonate (EMC) mixture (a commonly used Li-ion electrolyte solvent blend). All other solvent parameters (see Table 1) are for pure EC^32,33^.

**Table 1:** Solvent parameters for use in the SMD implicit solvent model.

Parameter	Meaning	Value
*ε*	Dielectric constant	18.5
*n*	Refractive index	1.415
$\sum {a}_{2}^{H}$	Abraham’s hydrogen-bond acidity	0.0
$\sum {\beta }_{2}^{H}$	Abraham’s hydrogen-bond basicity	0.735
*γ*	Relative surface tension	20.2
*ϕ*	Carbon aromaticity	0.0
*ψ*	Electronegative halogenicity	0.0

The dielectric constant *ε* represents a 3:7 weight blend of EC and EMC; all other parameters are for pure EC.

#### Error correction

Once a calculation has terminated, its output file is parsed for errors. If any errors are detected, then an empirically designed recipe-based error correction process is conducted. If the error handler recognizes the error and an appropriate remedy is available, then that remedy will be employed and the calculation will be restarted automatically, generally with some alteration to the input parameters. If an error is encountered in the re-started calculation, the same recipe-based error correction procedure is applied. If the error handler is unable to interpret the error, if all possible remedies have been exhausted, or if no remedy has been implemented for a particular error type, then the calculation fails.

Even if there are several possible remedies, only one remedy is applied at a time. The appropriate remedy for a given error may be sensitive to the parameters with which the calculation was run. Those parameters, in turn, may depend on the type and number of errors that the calculation has encountered previously.

**Fig. 3:**
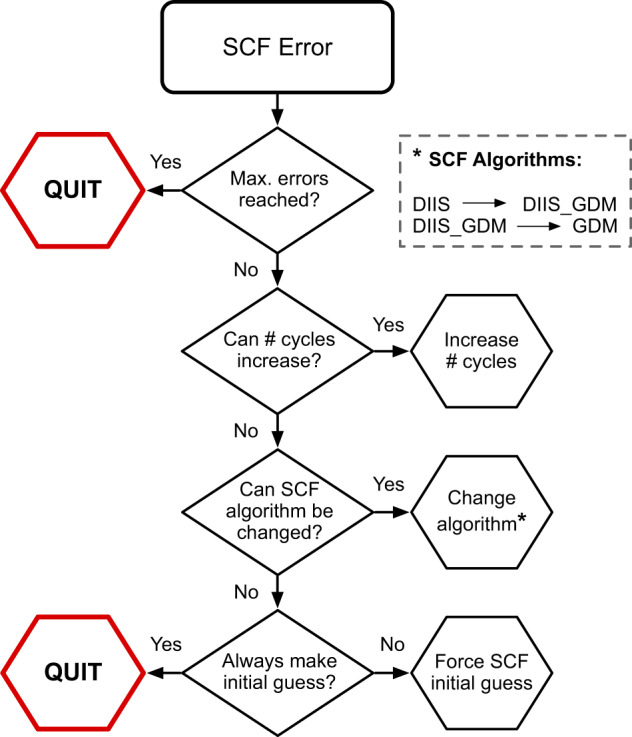
A flowchart for correcting an SCF convergence error. When the error is encountered, only a single remedy will be applied. If there is no possible remedy, or if too many errors have already been encountered, then the error handler will quit, and the calculation will be allowed to fail.

To illustrate the error-correction process, Fig. 3 depicts the logic dictating how a convergence error for a self-consistent field (SCF) calculation should be remedied. The first possible remedy involves increasing the number of SCF cycles allowed; if the number of SCF cycles is lower than some maximum value (typically 200), then the number of cycles are increased to that maximum. If that remedy cannot be applied, either because it has already been applied or because the user specified a large number of SCF cycles initially, then the next remedy is to alter the SCF algorithm. The geometric direct minimization (GDM) method^34^ tends to be highly robust at converging SCF calculations even for challenging molecules. However, because of its higher cost, the more rapid Direct Inversion of the Iterative Subspace method (DIIS)^35,36^ or a combination of the two methods (DIIS_GDM in Q-Chem) are used first, with GDM serving as a method of last resort. Finally, the SCF settings are altered such that an initial guess electron density is generated for each SCF calculation, with no knowledge of prior calculations. Using the previous solution as a starting point for an SCF calculation can improve efficiency, but it can also fail to capture electronic state reordering in a newly visited region of the PES, occasionally resulting in SCF convergence problems. If none of these remedies can be applied, if all of them have been applied already, or if the number of errors encountered in total has exceeded a user-defined limit (for this dataset, chosen as 5), then the calculation will fail without further attempt to remedy the error.

In addition to SCF convergence errors, remedies have been implemented for a range of errors that might arise during a calculation (failing to optimize the molecular geometry, failing to transform from internal to Cartesian coordinates, failing to calculate the Hessian eigenvalues for a vibrational frequency calculation, etc.) or while preparing a calculation (failing to parse the input file, failing to access the DFT code executable file, failure to access a license file, etc.).

### Convergence to potential energy surface minima

The goal of geometry optimization is to minimize the energy and to determine the stable molecular geometry. Generally, a optimizer will seek to reduce the gradient to zero, indicating that a stationary point has been found. However, convergence to a stationary point does not guarantee convergence to a local minimum of the PES; it is also possible to converge to an *n*th-order saddle point, where *n* is the number of imaginary frequencies. It is important to know when a calculation has converged to a saddle point and how to remedy it. Saddle points may provide poor approximations to the minimum energy structure and present significantly higher energy than the nearest minimum energy structure. Furthermore, saddle points can exhibit different bonding behavior from the minimum.

Most often, geometry optimization in DFT is conducted using a quasi-Newton-Raphson method; at each step, the energy and gradient are calculated, and the gradient is used to generate an approximation of the second derivative (Hessian) matrix^37^. While, in some methods, the exact Hessian is calculated at each step, this is prohibitively expensive in most cases and is therefore inappropriate for high-throughput applications. Because the Hessian used in quasi-Newton-Raphson optimization is not exact, the optimizer’s knowledge of the curvature of the PES is limited. This makes it relatively common for geometry optimization algorithms to converge to saddle points instead of minima, especially for complex reactive fragments and/or species in an implicit solvent environment.

**Fig. 4:**
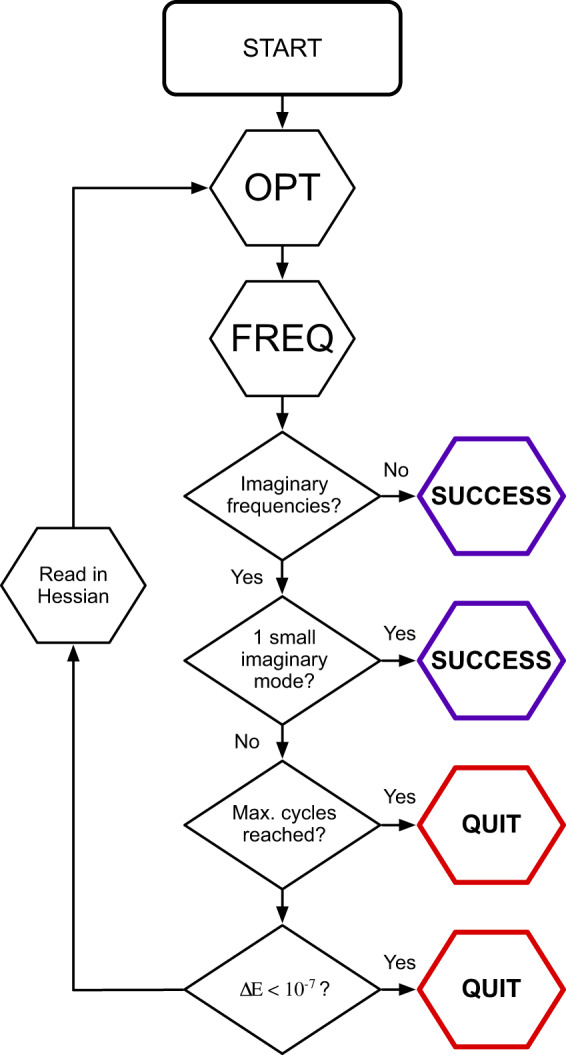
The frequency-flattening optimization (FFOpt) procedure. In the initial step, the geometry is optimized and a vibrational frequency calculation is performed. If there are no imaginary frequencies, or if there is a single imaginary frequency with very small magnitude, the calculation completes successfully. Otherwise, the Hessian from the vibrational frequency calculation will be used to inform the next cycle of optimization.

A method of “Frequency Flattening Optimization”, or FFOpt, is used to eliminate imaginary frequencies. As illustrated in Fig. 4, successive optimization calculations are conducted until the structure has converged to a true local minimum of the PES. In order to determine if a converged structure is a PES minimum or a saddle point, a vibrational frequency calculation is performed following each completed optimization calculation. Frequency calculations serve a dual purpose, simultaneously providing information about the curvature of the PES (the exact Hessian) and the nature of the converged stationary point while also providing some thermodynamic information, including the molecular enthalpy and entropy. If there are no imaginary frequencies, then the structure is confirmed to be a PES minimum, and no further calculations are needed. If there are imaginary frequencies, then the structure is a PES saddle point. The exact Hessian reported by the frequency calculation is then used as input to the subsequent optimization calculation in order to provide a better description of the local PES and allow the optimizer to move away from the saddle point and towards a true minimum. This procedure can be repeated as many times as needed until a minimum is found. We emphasize that the FFOpt procedure, like most geometry optimization methods, aims to optimize to a local minimum and does not guarantee convergence to the global minimum of the PES.

Here, in order to limit the computational cost of an individual calculation, no more than 10 frequency flattening cycles were allowed. Moreover, additional cycles will not be pursued if there is only one imaginary mode with a very small frequency magnitude (|*v*| ≤ 15 *cm*^−1^) or if the energy has changed by less than 10^−7^ Hartree (Ha) from the previous cycle to the current cycle, indicating that knowledge of the exact Hessian did not allow the optimization to leave the saddle point. Very small, singular imaginary frequencies are allowed because they may not correspond to true transition states; rather, they could be artifacts of numerical noise in the frequency calculation. If there is a single imaginary mode with a small frequency magnitude, the calculation is still considered a success; otherwise, a calculation which terminates with at least one imaginary frequency is considered a failure.

#### General calculation procedure

For a given set of unique molecular structures (as defined by the graph representations), FFOpt calculations were conducted at multiple charge states (−1, 0, and 1). For a particular charge state, when an even number of electrons was present, the molecule was initially assumed to be in a singlet state, and when an odd number of electrons was present, a doublet state was assumed.

Most commonly, low-spin states are preferred for molecular ground states, and stable high-spin states are rare^38^. This implies that one could expect that most molecules with even numbers of electrons should be in singlet states, rather than triplet states. However, triplets cannot be completely ignored, as there are exceptions (most notably diatomic oxygen) where triplet states are preferred at modest temperatures^39,40^. It is also possible that there exist species that are relevant to SEI formation which exhibit connectivity that can only exist as a triplet.

It would be computationally demanding to calculate all molecules and fragments in LIBE as both singlets and triplets. To balance computational cost and dataset diversity, only successfully optimized singlet molecules with less than 50 electrons were re-calculated as triplets. We note that this choice of cutoff is arbitrary, and there may be larger triplet species that are important to electrolyte or SEI formation reactions. Expanding the number of triplet species considered will be a future effort.

For all molecules for which the FFOpt procedure succeeded in identifying a PES minimum, a single-point calculation was conducted on the optimized geometry in order to produce a “cube” file of the electron density^41^. This cube file was then analyzed using Critic2 to determine the critical points and improve the determination of the molecular bonding.

Note that single-atom (Li, H, F, etc.) calculations use a different procedure. Because geometry optimization is unnecessary for such molecules, only single-point calculations to determine the energy and frequency calculations to determine the translational enthalpy and entropy components were conducted.

This general procedure of conducting FFOpt singlet and doublet calculations, selected triplet calculations, and finally single-point calculations, was used in several stages to build the LIBE dataset. These stages are described in detail below in the Dataset generation section.

### Dataset generation

#### Selection of principal molecules

The set of principal molecules was designed to adequately cover initial electrolyte molecules, experimentally identified SEI components, and other plausible intermediates or products that could arise during SEI formation in common LIB electrolytes. While many electrolyte chemistries have been developed for use in LIBs, the most widely used formulations involve a fluorinated salt such as lithium hexafluorophosphate (LiPF6)^42–46^, lithium bis(trifluoromethanesulfonyl)imide (LiTFSI)^20,47^, or lithium bis(flurosulfonyl)imide (LiFSI)^48,49^ dissolved in a solvent blend of cyclic carbonates such as ethylene carbonate (EC)^32,42^, or fluroethylene carbonate (FEC)^13,50–52^ and linear carbonates like dimethyl carbonate (DMC)^53,54^, diethyl carbonate (DEC)^55,56^, or ethyl methyl carbonate (EMC)^32,42^. At the current stage, LIBE contains molecules relevant to the electrolyte systems mentioned above (LiPF6, LiTFSI, LiFSI, EC, FEC, DMC, DEC, EMC) but does not, at this time, consider other electrolyte components or additives. However, we anticipate that the set will grow with more studies and applications. We note that we intend to incorporate all data in LIBE, and all future additions, in the Materials Project database^57,58^.

The general strategy for selecting principal molecules was as follows: a set of electrolyte molecules and non-polymeric SEI products related to those electrolytes were selected from the literature. In some cases (especially for products derived from EC), these molecules were then modified in two ways: hydrogen atoms and lithium atoms bonded to oxygen could be substituted for one another, and hydrogen atoms bonded to carbon could be replaced by fluorine. The former substitution was guided by proposed reaction pathways in which hydrogen fluoride can attack Li-O bonds to produce -OH groups and LiF; the latter modification was chosen because of the inclusion of FEC, which can participate in many similar reaction pathways as EC. No conformer searching was conducted on principal molecules; initial structures that minimized steric hindrance were posed by hand and optimized. During initial geometry optimization, there were some cases in which multiple conformers with different Li coordination environments were identified. In such cases, all identified conformers were accepted as distinct principal molecules.

Representations of all principal molecules are provided in Supplementary Table 1. These can be grouped into solvent molecules (Molecule numbers 1–13), salt molecules (14–16), inorganic SEI products (17–26), possible dissolved minority species, including gases (27–35), lithium ethylene dicarbonate (LEDC) and related derivatives (36–39), lithium butylene dicarbonate (LBDC) and related derivatives (40–47), lithium ethylene monocarbonate (LEMC) and related derivatives (48–60), ethanol and related derivatives (61–62), ethylene glycol (EG) and related derivatives (63–70), 1,4-butanediol and related derivatives (71–73), other molecules related to LiEC decomposition (74–77), and other molecules related to PF6- decomposition (78–87).

#### Molecular fragmentation

**Fig. 5:**
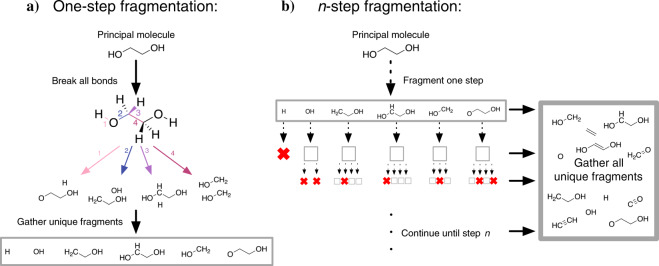
A schematic overview of a molecular fragmentation process. For a single-step fragmentation (**a**), a principal molecule is selected (in this case, principal molecule 70 in Supplementary Table 1). Each bond is broken, generating a collection of molecular fragments. These fragments are then filtered to generate a set of unique (non-isomorphic) molecules. In an n-step fragmentation (**b**), this process is repeated in a recursive fashion. At each step, all fragments from the previous step are collected and undergo a single-step fragmentation. If the fragment is a single atom with no bonds or if all fragments generated are already present in the collection, then the process terminates (red “X”). When the maximum number of steps has been reached, or when no new fragments can be generated, the n-step fragmentation terminates.

Fragmentation begins with the molecular graph representation and 3D structure of a molecule of interest. In a single fragmentation step (Fig. 5a), each individual bond in the molecular graph is broken, generating either one or two fragments. In the case of a single fragment - indicating that the bond was part of a ring - an initial structure for the ring-opened fragment was generated using a low-cost optimization with the UFF force field as defined in OpenBabel. This preliminary optimization was conducted with the aim of preventing the ring from immediately re-closing during geometry optimization. In the case of two fragments, the coordinates associated with the atoms in each fragment were used as the initial structure. After all bonds have broken, all unique fragments - defined by their graph representations - were collected.

In most cases, it was desirable to not only obtain the products of single-bond cleavage, but all possible sub-fragments of a given molecule. This can be done by recursively applying the above single-step fragmentation method (Fig. 5b). At the *n*th step, all new structures from the *n−*1th step undergo a single-step fragmentation if possible (single atoms, which have no bonds, cannot be fragmented); the final set of fragments at that step is the union of the sets of unique fragments from each such single-step fragmentation. This recursive fragmentation can be continued until all fragments contain no bonds (at which point until only single atoms remain).

Supplementary Table 1 includes the number of fragmentation steps allowed for each principal molecule. In most cases, the number of steps was chosen such that all possible bonds were broken, indicated with “MAX”. For larger molecules (with 20 or more atoms), computing the properties of all possible sub-fragments would be too computationally costly, hence a smaller number of steps was used. After fragmenting each principal molecule using the appropriate number of steps, all unique fragments - again defined by graph connectivity - were analyzed using DFT.

#### Generation of recombinant molecules

Reactions in LIB electrolytes involve not only bonds being broken but also bonds being formed. The set of principal molecules includes known products, implicitly accounting for some bond formations between possible fragments. However, fragmenting these principal molecules does not guarantee that all important intermediates or even all products are included. To improve the coverage of possible intermediate and product species and set the stage for new knowledge of SEI formation, some fragments (see criteria below) were allowed to recombine to form new molecules.

**Fig. 6:**
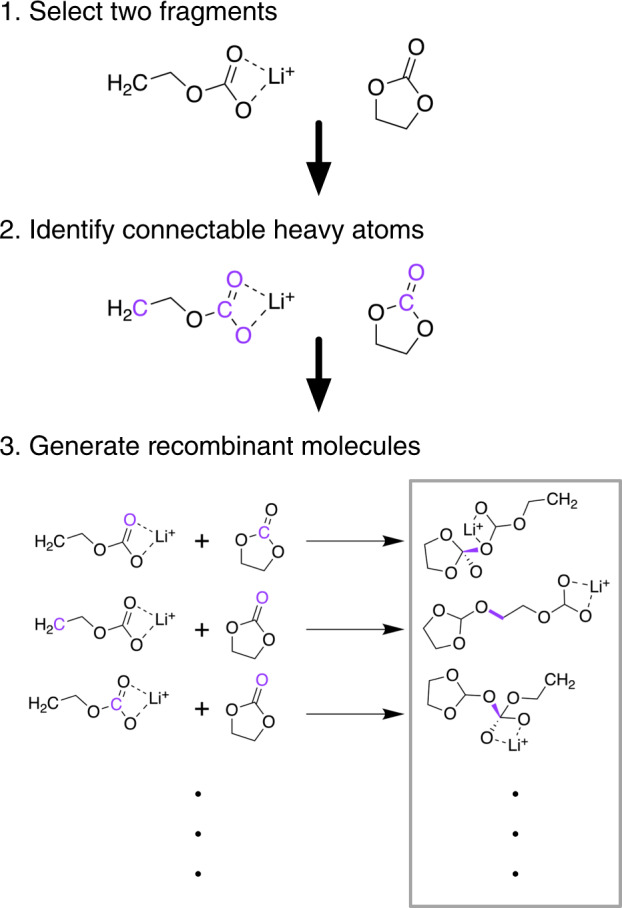
A simplified depiction of the recombination process. First (1), two fragments - in this case, from lithium ethylene carbonate, or principal molecule 1 - are selected. The heavy atoms in these molecules that can form additional bonds (shown in purple) are identified using valence rules (2), and finally, bonds (also in purple) are added between all combinations of these connectable heavy atoms (3) to form a set of unique recombinant molecules (gray box).

After all fragment species had been analyzed using DFT, a subset were selected for recombination. Specifically, all fragments from a two-step fragmentation of LiEC (principal molecule 1 in Supplementary Table 1) that could be formed exergonically from LiEC were included, as well as all fragments of H2O. All combinations of two fragments were recombined by adding a single bond in all possible ways that respect the typical valence rules of different atoms (Fig. 6). For instance, if one fragment has an oxygen connected to only one atom and one fragment has a carbon connected to only three atoms, then they would be allowed to combine. On the other hand, that same oxygen would not be allowed to combine with a carbon connected to four atoms. In applying these bonding rules, we do not count metal coordinate bonds and do not consider bond order (a single bond is treated on the same footing as a double or triple bond), but only consider the number of non-metal atoms connected to a given atom.

The recombinant molecules generated in this manner were further filtered by considering the reaction free energies of the recombination reactions. The BonDNet neural network was employed to predict the bond formation energies of the recombinant molecules. If the formation of the bond is predicted to be endergonic (the recombinant molecule was less stable than the constitutent fragments), then the recombinant molecule was discarded. Initial guess structures of all remaining molecules were produced using the OPLS_2005 force field^59^ as implemented in the Schrödinger Suite^60^, and these initial structures were analyzed using DFT.

Note that the numerous stages of filtering used here - beginning with a small number of fragment molecules, requiring valence rules to be obeyed, and screening by bond formation energy - are necessary to limit the number of recombinant molecules considered. Recombination, as described here, is an inherently combinatorial process. Without appropriate filters, massive numbers of recombinant molecules can be generated, far too many to be calculated using high-accuracy DFT methods. We estimate that, if we attempted to recombine all fragment molecules included in LIBE, we would generate over 2,000,000 new molecules, which is completely intractable at our chosen level of theory. Future work will include efforts to recombine fragments of a larger set of principal molecules, most importantly salt species.

### Final analysis

Molecular enthalpies, entropies, and free energies at 298.15K were calculated in multiple ways. The raw electronic energies, enthalpies, and entropies calculated in Q-Chem were used and are provided in the given units (Ha for electronic energy, kcal·mol^−1^ for enthalpy, and cal·mol^−1^·K^−1^ for entropy), as well as in eV (or eV·K^−1^ for entropy). In addition, two different methods to correct for errors in the rigid-rotor harmonic oscillator (RRHO) approximation (used in Q-Chem) are employed: that of Ribiero *et al*.^61^, in which low-frequency vibrational modes are shifted to some higher frequency (100 cm^−1^) and that of Grimme^62^, in which low-frequency modes are treated not as vibrations but as rotations. In all cases, imaginary frequencies are ignored for the purposes of calculating enthalpy, entropy, and free energy.

The point groups of all molecules were identified using the PointGroupAnalyzer tool implemented in pymatgen^63^.

## Data Records

Data for 17,190 molecules generated using an *ω*B97X-V/def2-TZVPPD/SMD level of theory are provided in a Figshare repository^64^. The data, including optimized 3D coordinates, partial charges and spins (from Mulliken population analysis^65^, the Restrained Electrostatic Potential (RESP) method^66^, and Critic2), molecular connectivity information, vibrational information (frequencies, vibrational mode vectors, IR intensities), and thermodynamic quantities (energy, enthalpy, entropy, Gibbs free energy), are contained in a single JSON-formatted file, libe.json. Molecules for which calculations failed or which otherwise failed the tests described in the Technical Validation section are not included in this collection.

Table 2 describes the keys in each entry of the libe.json file. Note that in some cases, keys may have no associated value; for instance, a single atom has no bonds.

**Table 2:** Description of keys present in LIBE dataset entries.

Key	Description
molecule_id	Unique identifier (format: libe-XXXXXX, where XXXXXX is a 6-digit number
bonds	List of pairs (*a*, *b*), where *a* and *b* are the 0-based indices of bonded atoms
charge	Charge of the molecule
chemical_system	Collection of elements present (ex: “C-H-O” for a molecule with C, H, and O present)
composition	Keys are elements; values are the number of atoms of those elements present
elements	List of elements present
formula_alphabetical	Simple chemical formula, with elements in alphabetical order (ex: “C4 H8 O1”)
molecule	Serialized pymatgen Molecule object, containing species, coordinates, charge, and spin multiplicity
molecule_graph	Serialized pymatgen MoleculeGraph object; molecule with graph representation
number_atoms	Number of atoms in the molecule
number_elements	Number of unqiue elements present in the molecule
partial_charges	Atomic partial charges, calculated using various methods (Mulliken, RESP, Critic2)
partial_spins	For open-shell molecules, atomic partial spins, calculated with Mulliken population analysis
point_group	Molecular point group in Schönflies notation
species	Elements present at each atom in the molecule, in order
spin_multiplicity	Spin multiplicity (2*S* + 1) of the molecule
thermo	Molecular thermodynamics, calculated from Q-Chem or a modified RRHO method
vibration	Calculated vibrational frequencies, and associated vibrational mode vectors and IR intensities
xyz	3D coordinates of the atoms in the molecule, in same order as “species”

## Technical Validation

### Level of theory

In order to maximize the utility of the LIBE dataset, a relatively costly but accurate level of theory was chosen. In an extensive benchmark study of density functionals by Mardirossian and Head-Gordon^67^, *ω*B97X-V was found to be the most suitable hybrid generalized gradient approximation (hybrid GGA) functional, with exceptional accuracy for bonded interactions and noncovalent interactions. It is worth noting that *ω*B97X-V also displays high accuracy for calculation of barrier heights; while no transition states are included in LIBE, this is still beneficial, as it implies that the kinetic properties of reactions between molecules within the dataset could be reliably calculated without modification to the level of theory. While, to the best of our knowledge, no benchmark study has systematically examined how *ω*B97X-V performs for calculations involving charged, radical, and metal-coordinated species in solution, *ω*B97X-V has been shown to exhibit exceptional performance for calculations involving transition metal complexes^68^ and metal-organic reactions^69^ in gas phase. Additionally, a previous *ab initio* molecular dynamics study^70^ found that *ω*B97X-V was able to model aqueous solutions of NaCl more accurately than most density functionals, producing results in qualitative agreement with experiment. The benchmark study by Mardirossian and Head-Gordon found using a limited set of density functions that the def2-TZVPPD basis set performed nearly as well as the much larger def2-QZVPPD basis set^67^, which makes it especially useful for high-throughput studies involving many thousands of calculations.

Generally, it should be expected that the use of an implicit solvation model should improve the accuracy of calculations involving molecules in solvent. Specifically, the SMx family of models, including the SMD model shown here, have been shown to accurately predict solvation free energies^29,71,72^ as well as redox potentials^73^, improving upon the more simple PCM models due to their inclusion of non-electrostatic effects.

We also justify our choice of level of theory by noting that similar levels of theory have previously been used to generate datasets used to study reactivity. In particular, Grambow *et al*.^74^ recently used the *ω*B97X-D3 density functional^75^ (which is closely related to *ω*B97X-V and differs primarily in the choice of dispersion correction) and the def2-TZVP basis set (which is part of the same family as def2-TZVPPD but contains no diffuse functions and fewer polarization functions) to create a dataset of over 12,000 organic reactions (including optimized reactants, products, and transition states) in vacuum. The solution-phase charged and radical organometallic chemistry involved in SEI formation is more complex than the gas-phase organic reactions considered by Grambow *et al*., necessitating both the inclusion of an implicit solvent model and the use of a larger basis set including diffuse functions.

### Data filtering

We note that the error correction procedures that we have employed are successful in decreasing the likelihood of failure in FFOpt calculations. Without intervention, roughly 25% of all calculations fail due to an error (for instance, inability to achieve a converged SCF solution or an inability to optimize a molecular geometry in the allowed number of steps), encounter a significant imaginary frequency (with magnitude > 15 cm^−1^), or optimize to a structure with multiple disconnected fragments. With our error-handling procedures employed, this failure rate drops below 5% on average. In cases where error correction procedures were unable to eliminate issues, the calculations were not included in LIBE. While, in principle, single-point and Critic2 calculations could also be a source of failure, in practice such calculations almost never failed when applied to optimized molecular structures.

Of the successful calculations that produced PES minima with connected structures, additional filters were put in place to ensure data quality and prevent duplicate molecules from being included in LIBE. First, molecules were eliminated if the energy of the molecule at the end of the geometry optimization differed from the energy calculated from the subsequent single-point calculation by more than 0.001 Hartree. Such a disagreement in energy implies that the single-point calculation converged to a different minimum of the electron density than was found at the end of the geometry optimization, potentially leading to inaccurate or inconsistent determination of bonding or atomic partial charges. Additionally, duplicate molecules were removed from the dataset. If two or more sets of calculations produced molecules that were non-equivalent (had different 3D coordinates) but with identical bonding, charge, and spin multiplicity, then only the molecule with the lowest calculated electronic energy was included in LIBE. Note that, while we did not explicitly perform any conformer searches, this filter implicitly selects the lowest-energy conformer that had been calculated.

### Dataset diversity

The LIBE dataset is designed for the study of (electro)chemical reactivity in LIB. As such, the most important consideration is whether the dataset adequately captures the possible molecules that could form in a LIB as a result of electrolyte decomposition. Considering that many common electrolyte molecules and most reported non-oligomeric/non-polymeric products derived from those molecules are among the principal molecules used to generate LIBE, we believe this is the case.

**Fig. 7:**
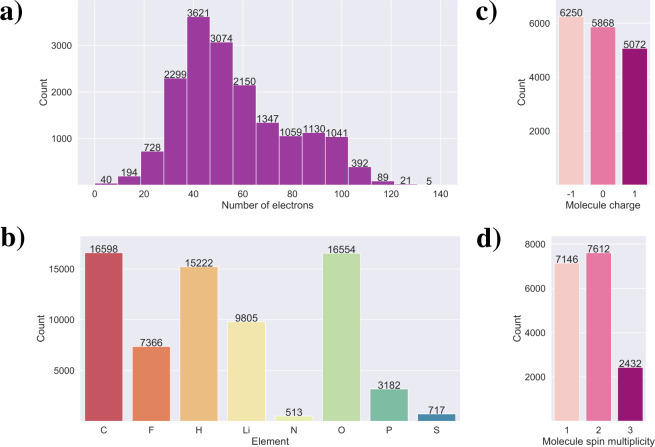
An analysis of the composition of the LIBE dataset in terms of: number of molecules with different numbers of electrons (**a**); number of molecules with various elemental species (**b**); number of molecules with charges -1, 0, and 1 (**c**); and number of molecules with spin multiplicity 1, 2, and 3 (**d**).

For uses outside of this domain, it is worth examining the chemical diversity of the LIBE dataset. While the dataset skews towards small molecules by design (both because most molecules examined are fragments of larger molecules and because large molecules would be computationally expensive), Fig. 7a shows that the distribution of molecules by size (measured by number of electrons) is wide; similar distributions are found when the size is measured by number of atoms and number of bonds.

Because most principal molecules are organic in nature and more specifically are derived from lithiated organic carbonates, the dataset is biased towards the C-H-O-Li chemical system, though with many (7,366) fluorine-containing molecules present as well (see Fig. 7b). While many phosphorus-containing molecules (3,182) are included, the bonding motifs (see Table 3) observed for phosphorus are limited (only F-P, O-P, and a small number of C-P, H-P, and Li-P bonds are present) because these molecules are all derived from PF6 and related molecules. We further note that the diversity in nitrogen- and sulfur-containing species is lacking because they are present only from TFSI- and FSI-based fragments.

**Table 3:** Number of different types of bonds present in the LIBE dataset.

Bond Type	Number of Bonds
C-C	25,744
C-F	6,002
C-H	53,178
C-Li	2,626
C-N	129
C-O	55,186
C-P	256
C-S	636
F-F	10
F-H	74
F-Li	1,285
F-O	150
F-P	4,604
F-S	195
H-H	4
H-Li	9
H-O	4,266
H-P	21
Li-Li	1
Li-N	53
Li-O	18,821
Li-P	19
Li-S	89
N-O	28
N-S	867
O-O	346
O-P	4,925
O-S	1,387
S-S	34

While there are similar numbers of neutral molecules (5,868) and molecules with charge −1 (6,250), there are somewhat fewer molecules with charge +1 (5,072) (Fig. 7c). Because calculations were attempted for all initial molecule structures at charges −1, 0, and +1, this implies that the cationic species were more likely to fail than the anions or neutral species. There are slightly more doublet species (7,612) than singlets (7,146) (Fig. 7d). As discussed above, the number of triplets was intentionally kept low to reduce computational cost. We note that of the 1,961 pairs where singlet and triplet calculations optimized to isomorphic structures, the triplet was lower in electronic energy in 11.98% (235) of cases. Further, there are 471 triplet molecules for which no isomorphic singlet with the same charge exists. Thus, it is possible that some number of unique structures, and some stable ground-states for existing structures, may be missing from LIBE. Because most often, the singlet structure is more stable than the triplet structure in the ground state, this lack of triplets should not be a significant detriment to the quality of the data.

## Usage Notes

The libe.json file provided can be analyzed by any code capable of parsing JSON documents. The “molecule” and “molecule_graph” keys (see Table 2) are JSON representations of Python objects, and so Python-based analysis tools may be most convenient; however, the data stored in these objects is redundant, so this choice is not necessary.

**Fig. 8:**
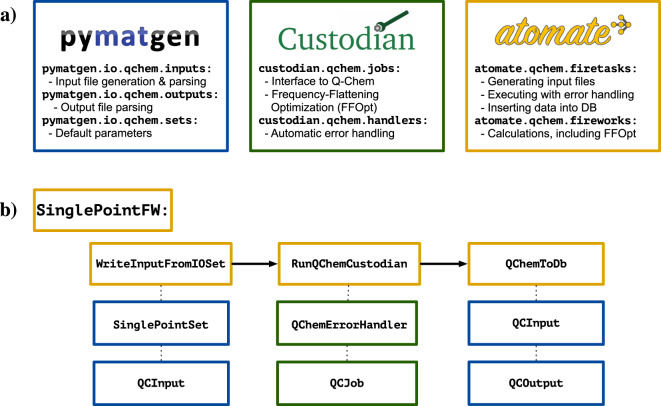
An overview of our automated high-throughput molecular DFT framework, as implemented in pymatgen (blue), custodian (green), and atomate (yellow) (**a**); an example calculation (Firework) for geometry optimization (**b**), indicating the different steps and the ways in which pymatgen, custodian, and atomate interact. First, the input file is written using default parameters defined in pymatgen. Then, the geometry optimization calculation is performed using the Q-Chem interface in custodian and an automated error handler. Finally, once the calculation is finished, the input and output files are parsed using pymatgen, and the results from the calculation are added to a database.

We have created a software repository, deliberate, to aid in the use and analysis of the LIBE dataset. It includes the following files:

- plotting.py: Contains a utility function for making categorical bar plots and histograms
- filters.py: Contains functions for filtering the dataset
- recombination.py: Contains some basic code for molecular recombinantion
- data_generation.ipynb: A Jupyter Notebook providing a basic example of a fragmentation and recombination scheme to generate molecules from an initial set of principal molecules
- dataset_composition.ipynb: A Jupyter Notebook analyzing the composition of the LIBE dataset in some basic dimensions (bond types, molecule charge, molecule spin multiplicity, etc.)
- filters.ipynb: A Jupyter Notebook employing the filters in filters.py, which might be useful to tailor the dataset to a particular application.

## Supplementary information

Supplementary Table 1

## Data Availability

Our computational infrastructure for high-throughput and automated DFT calculations using the Q-Chem electronic structure code is implemented in existing open-source Python packages developed by the Materials Project, namely pymatgen^63^, custodian, and atomate^76^. The modules in these codes used specifically for Q-Chem, along with their purposes, are described in Fig. 8a. The basic functionality to generate, process, analyze, and manipulate molecules is included in pymatgen. We have added functionality to read and write Q-Chem input files and to parse Q-Chem output files. In addition, we have developed a number of “Sets”, pre-defined collections of input parameters appropriate for common types of calculations. While these sets can be used with any level of theory available in Q-Chem, it is especially facile to use the advanced level of theory used for the LIBE dataset (*ω*B97X-V/def2-TZVPPD/SMD). The custodian Q-Chem module defines the interface between Q-Chem and our automation framework in atomate. It can execute arbitrary Q-Chem jobs and can automatically check for, detect, and correct errors in Q-Chem calculations. custodian also handles the logic for FFOpt calculations. The Q-Chem module in atomate combines the Q-Chem input and output modules in pymatgen and the Q-Chem interface and error handlers in custodian to perform Q-Chem jobs and analyze their data in a high-throughput fashion. An example calculation, or Firework, for a single-point optimization is shown schematically in Fig. 8b. First, based on some input parameters, a Q-Chem input file for a geometry optimization calculation is written. Then, the optimization job is run, with custodian waiting for completion and, upon completion, checking for errors. If the job completes without errors, then the output is parsed and stored in a database. Individual Q-Chem calculations, represented in atomate by Fireworks like SinglePointFW, can be combined to form more complex workflows. Other than Q-Chem itself, all the necessary code used to generate and analyze the LIBE dataset (pymatgen: http://github.com/materialsproject/pymatgen; custodian: http://github.com/materialsproject/custodian; atomate: http://github.com/hackingmaterials/atomate; and deliberate: http://github.com/espottesmith/deliberate) can be found on Github.
