# Supplementary material for: Quantum chemical calculations of lithium-ion battery electrolyte and interphase species
Source: Sci Data. 2021 Aug 5;8:203. doi: 10.1038/s41597-021-00986-9 (PMC8342431; doi:10.1038/s41597-021-00986-9)
Supplement: Supplementary file 1 — Supplementary Table 1 [file 41597_2021_986_MOESM1_ESM.pdf]

Supplementary Table 1 for: Quantum chemical calculations of lithium-ion battery electrolyte and interphase species

Molecule Number	Structure	Fragmentation Steps
1	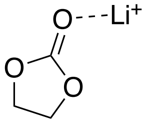	MAX
2	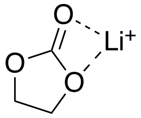	MAX
3	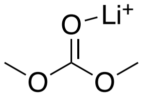	MAX
4	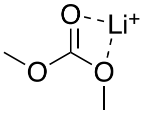	MAX
5	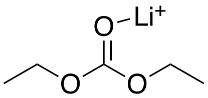	MAX
6	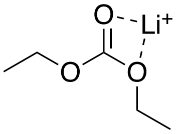	MAX
7	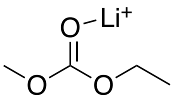	MAX
8	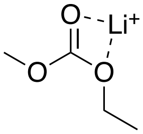	MAX
9	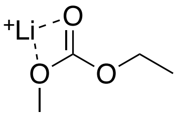	MAX

10	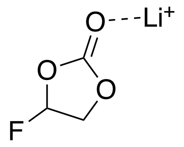	MAX
11	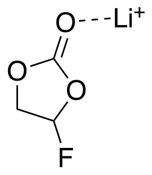	MAX
12	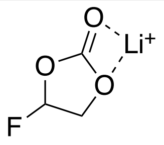	MAX
13	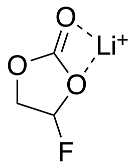	MAX
14	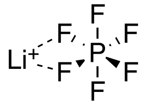	MAX
15	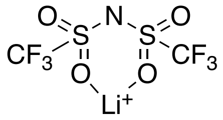	MAX
16	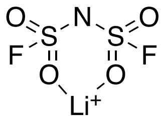	MAX
17	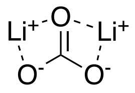	MAX
18	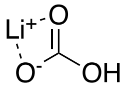	MAX
19	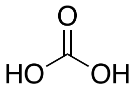	MAX
20	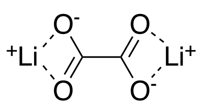	MAX

21	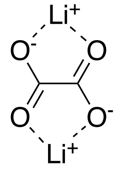	MAX
22	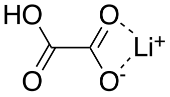	MAX
23	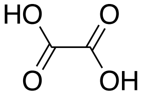	MAX
24	Li–OH	MAX
25	Li–O–Li	MAX
26	Li–F	MAX
27	O_2	MAX
28	H_2	MAX
29	F_2	MAX
30	O=C=O	MAX
31	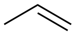	MAX
32	FCH_2CH_3	MAX
33	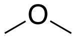	MAX
34	H_3O	MAX
35	HF	MAX
36	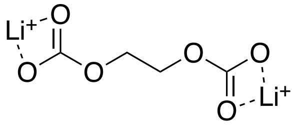	MAX
37	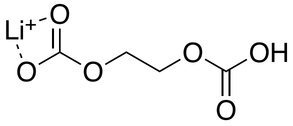	MAX
38	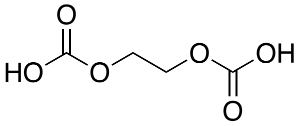	MAX
39	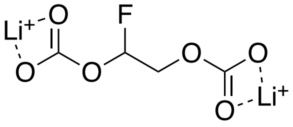	MAX

40	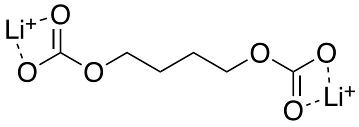	3
41	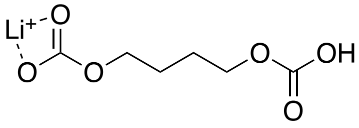	3
42	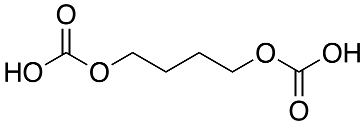	3
43	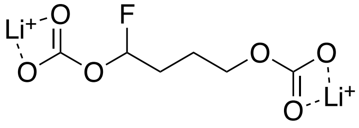	2
44	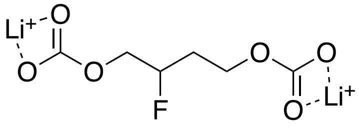	2
45	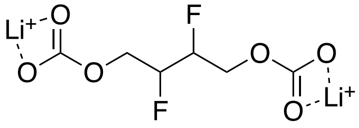	2
46	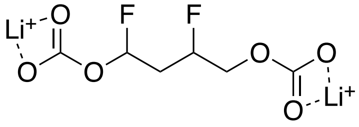	2
47	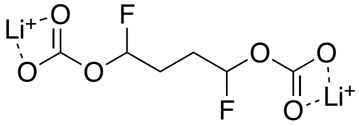	2
48	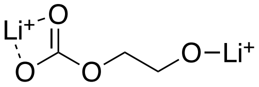	MAX
49	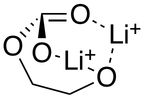	MAX
50	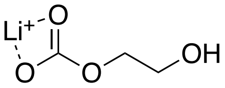	MAX
51	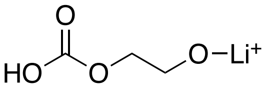	MAX

52	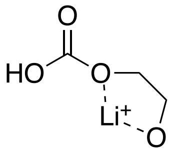	MAX
53	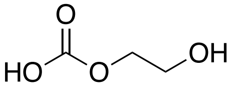	MAX
54	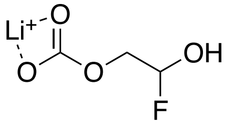	MAX
55	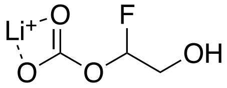	MAX
56	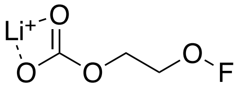	MAX
57	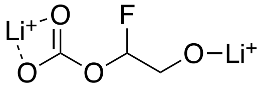	MAX
58	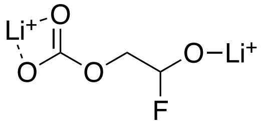	MAX
59	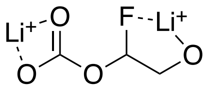	MAX
60	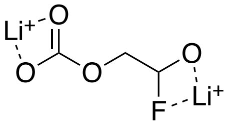	MAX
61	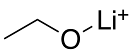	MAX
62	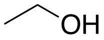	MAX
63	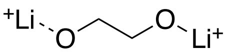	MAX
64	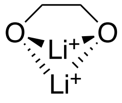	MAX
65	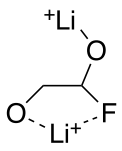	MAX

66	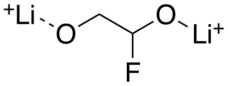	MAX
67	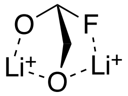	MAX
68	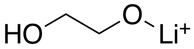	MAX
69	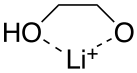	MAX
70	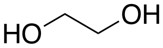	MAX
71	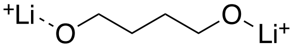	MAX
72	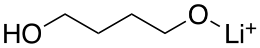	MAX
73	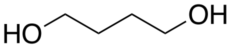	MAX
74	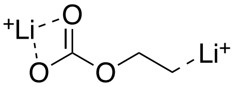	MAX
75	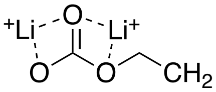	MAX
76	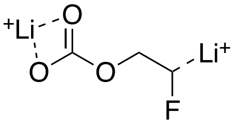	MAX
77	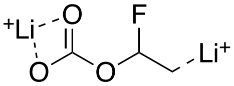	MAX
78	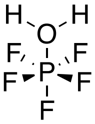	MAX
79	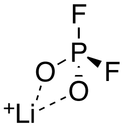	MAX
80	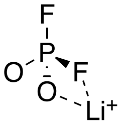	MAX
81	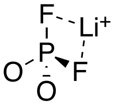	MAX

82	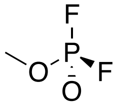	MAX
83	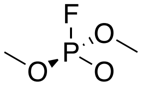	MAX
84	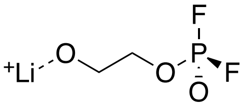	MAX
85	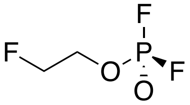	MAX
86	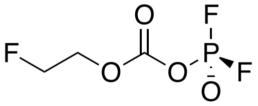	MAX
87	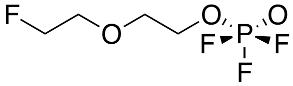	4

Table 1: Principal molecules used for fragmentation, including depth of fragmentation. A fragmentation depth of "MAX" indicates that all possible combinations of bonds were broken during fragmentation.
